# The Possible Role of Tumor Necrosis Factor-*α* in Diabetic Polyneuropathy

**DOI:** 10.1155/EDR.2003.65

**Published:** 2003

**Authors:** Jo Satoh, Soroku Yagihashi, Takayoshi Toyota

**Affiliations:** 1 Division of Molecular Metabolism and Diabetes Department of Internal Medicine Tohoku University Graduate School of Medicine 2-1 Seiryo-machi, Aoba-ku Sendai 980-8575 Japan; 2 The First Department of Pathology Hirosaki University School of Medicine Hirosake Japan; 3 Tohoku Rosai Hospital Sendai Japan

## Abstract

In this review, the authors provide evidences that imply the role of tumor necrosis factor-*α* (TNF-*α*) in the pathogenesis of diabetic complications, especially diabetic polyneuropathy. Under chronic hyperglycemia, endogenous TNF-*α* production is accelerated in microvascular and neural tissues, which may undergo an increased microvascular permeability, hypercoagulability, and nerve damage, thus initiating and promoting the development of characteristic lesions of diabetic microangiopathy and polyneuropathy. Enhanced TNF-*α* production may also promote atherosclerosis due to increased insulin resistance and the expression of adhesion molecules. Clinical application of specific agents that suppress production and/or activity of TNF-*α* may inhibit the development and exacerbation of chronic diabetic complications.


*Experimental Diab. Res.*, 4:65–71, 2003
Copyright © Taylor and Francis Inc.
ISSN: 1543-8600 print / 1543-8619 online
DOI: 10.1080/15438600390228496

Taylor & Francis
Taylor & Francis Group

REVIEW

### The Possible Role of Tumor Necrosis Factor-$\alpha$ in Diabetic Polyneuropathy

**Jo Satoh,[1] Soroku Yagihashi,[2] and Takayoshi Toyota[3]**

[1]*Division of Molecular Metabolism and Diabetes, Department of Internal Medicine, Tohoku University Graduate School of Medicine, Sendai, Japan*
[2]*The First Department of Pathology, Hirosaki University School of Medicine, Hirosake, Japan*
[3]*Tohoku Rosai Hospital, Sendai, Japan*

**In this review, the authors provide evidences that imply the role of tumor necrosis factor-$\alpha$ (TNF-$\alpha$) in the pathogenesis of diabetic complications, especially diabetic polyneuropathy. Under chronic hyperglycemia, endogenous TNF-$\alpha$ production is accelerated in microvascular and neural tissues, which may undergo an increased microvascular permeability, hypercoagulability, and nerve damage, thus initiating and promoting the development of characteristic lesions of diabetic microangiopathy and polyneuropathy. Enhanced TNF-$\alpha$ production may also promote atherosclerosis due to increased insulin resistance and the expression of adhesion molecules. Clinical application of specific agents that suppress production and/or activity of TNF-$\alpha$ may inhibit the development and exacerbation of chronic diabetic complications.**

**Keywords** Diabetic Neuropathy; Gliclazide; *N*-acetylcysteine (NAC); TNF-$\alpha$; TNF-$\alpha$ Promoter Gene Polymorphism; TNF-$\alpha$ Suppressants; Troglitazone

Tumor necrosis factor-$\alpha$ (TNF-$\alpha$) was originally discovered as a monokine produced by macrophages. It was subsequently revealed that various cells, such as fibroblasts, epithelial cells, adipocytes, and myocytes, also produce TNF-$\alpha$, which has a variety of biological activities. It has been indicated that TNF-$\alpha$ plays a role in the pathogenesis of not only type 1 diabetes mellitus [1] but also type 2 diabetes mellitus [2]. Recent studies have further disclosed that this peptide contributes to the development of diabetic complications [3, 4]. In this review, we propose a hypothesis on a possible role of TNF-$\alpha$ in chronic diabetic complications, especially in diabetic polyneuropathy.

### INCREASED PRODUCTION OF TNF-$\alpha$ IN A DIABETIC STATE

During the study on the role of TNF-$\alpha$ in type 1 diabetes [5], we incidentally found that lipopolysaccharide (LPS)-induced serum TNF-$\alpha$ significantly increased in vivo in an animal model of diabetes after development of diabetes, irrespective of the type of diabetes, i.e., BB rats and NOD mice for a model of type 1 diabetes and GK rats for a model of type 2 [6]. Serum TNF-$\alpha$ levels were also higher in patients with type 2 diabetes than those in nondiabetic patients [7]. Thus, in vivo production of TNF-$\alpha$ is increased under chronic hyperglycemia. The mechanisms of enhanced TNF-$\alpha$ production may be ascribed to macrophage stimulation by high glucose itself [8], hyperglycemia-induced oxidative stress [9], or exposure to advanced glycation end products (AGEs) [10]. The increased production of TNF-$\alpha$ in vivo may exacerbate insulin resistance [11] and eventually promote diabetic complications as discussed below.

Received 9 May 2003; accepted 14 May 2003.
This research was supported by a Grant-in-Aid for Scientific Research (C) from The Ministry of Education, Science, Sports and Culture of Japan (J.S.), a grant from the Japan Diabetes Foundation (J.S.), and Takeda Medical Research Foundation (J.S.).
Address correspondence to Jo Satoh, Division of Molecular Metabolism and Diabetes, Department of Internal Medicine, Tohoku University Graduate School of Medicine, 2-1 Seiryo-machi, Aoba-ku, Sendai, 980-8575, Japan. E-mail: jsatoh@int3.med.tohoku.ac.jp

### TNF-$\alpha$ AND TYPE 2 DIABETES

### TNF-$\alpha$ and Insulin Resistance

Increased TNF-$\alpha$ production secondary to hyperglycemia may be a factor that exacerbates insulin resistance in poorly controlled diabetes because TNF-$\alpha$ induces insulin resistance [2, 11]. Hotamisligil and Spiegelman reported that TNF-$\alpha$ mRNA and protein expression increases in the adipocytes of obese animals and TNF-$\alpha$ induces insulin resistance in the muscles and the adipocytes themselves in paracrine and autocrine fashions [2]. The molecular mechanisms by which TNF-$\alpha$ induces insulin resistance are considered to be the following. TNF-$\alpha$ binds TNF receptor 1 and activates sphingomyelinase that metabolizes sphingomyelin to ceramide [12]. Ceramide increases serine phosphorylation of insulin receptor substrate-1 (IRS-1), which inhibits the insulin receptor tyrosine phosphorylation, resulting in attenuation of insulin signaling and a decrease in glucose transporter-4 (GLUT-4) translocation and glucose uptake. TNF-$\alpha$–mediated insulin resistance associated with obesity may be related to metabolic syndrome [2]. Insulin resistance related to TNF-$\alpha$ in obese animals has also been reported in obese humans [13].

### TNF-$\alpha$ and Chronic Diabetic Complications

Chronic hyperglycemia activates macrophages [8–10] and stimulates in vivo TNF-$\alpha$ production [6, 7]. Enhanced TNF-$\alpha$ production in a diabetic state may promote the development of diabetic micro- and macroangiopathies through a variety of TNF-$\alpha$ bioactivities. For example, TNF-$\alpha$ increases the permeability of the endothelium through release of nitric oxide [14] and increases thrombogenesis through plasminogen activator inhibitor-1 (PAI-1) overexpression [15]. A role of TNF-$\alpha$ in angiopathies is supported by a report in which cerebral ischemia is reduced by neutralizing serum TNF-$\alpha$ with specific antibody in spontaneously hypertensive rats [16]. Furthermore, TNF-$\alpha$ stimulates the expression of adhesion molecules on the endothelial cells [17]. The serum levels of free adhesion molecules (vascular cell adhesion molecule-1, VCAM-1) significantly correlate with the intima-media complex thickness (IMT) of the carotid artery [18]. These imply that TNF-$\alpha$ accelerates atherosclerosis by inducing the expression of adhesion molecules on the endothelial cells.

### SUPPRESSION OF DIABETIC COMPLICATIONS BY INHIBITING TNF-$\alpha$ PRODUCTION

Based on the hypothesis that TNF-$\alpha$ is also implicated in microvascular complications in diabetes, we administered TNF-$\alpha$ to diabetic rats and measured the motor nerve conduction velocity (MNCV) of the sciatic nerve to explore the effect of TNF-$\alpha$ on nerve function. Administration of TNF-$\alpha$ significantly decreased MNCV in diabetic rats (Figure 1), although it did not influence the MNCV in nondiabetic rats [19]. This finding strongly implies that TNF-$\alpha$ contributes to diabetic nerve dysfunction and indicates that suppression of enhanced TNF-$\alpha$ production in a diabetic state might possibly attenuate the progression of diabetic polyneuropathy. To test this hypothesis, we performed experiments using some reagents known to inhibit TNF-$\alpha$ production [20–23].

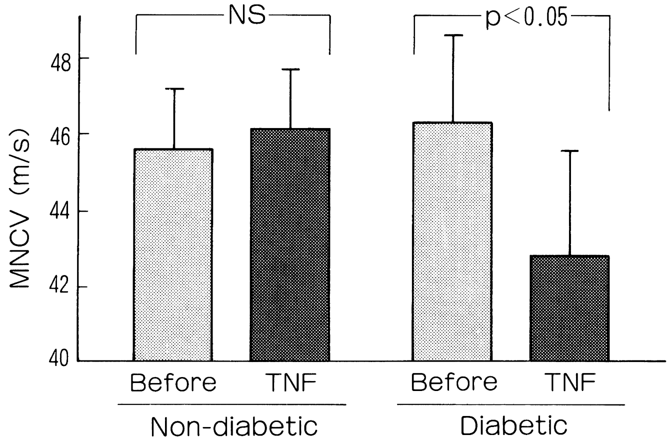

**FIGURE 1**
Suppression of motor nerve conduction velocity by TNF-$\alpha$ administration in diabetic rats. Streptozotocin-induced diabetic rats and nondiabetic rats were administered $1 \times 10^5$ units of recombinant human TNF-$\alpha$ and 6 hours later MNCV of the tibial nerve was measured [19].

### Inhibition of Experimental Diabetic Neuropathy with *N*-acetylcysteine (NAC)

NAC is a precursor of glutathione, which is important in the redox reaction in cells and is clinically used as an antioxidant for detoxication [24]. NAC is a free-radical scavenger that has been reported to suppress TNF-$\alpha$ production in vitro and in vivo [25]. We could reproduce the inhibitory effect of NAC on TNF-$\alpha$ production in diabetic rats [26]. Lipopolysaccharide (LPS)-induced serum TNF-$\alpha$ levels were significantly increased in streptozotocin-induced diabetic rats as compared with those in nondiabetic control rats. In diabetic rats, glucose levels were maintained at >300 mg/dL (16.7 mM) for 12 weeks of the experimental period. Although daily oral administration of NAC did not alter blood glucose levels, it dose-dependently inhibited LPS-induced serum levels of TNF-$\alpha$ in diabetic rats [26].

Next, we observed the effect of NAC on MNCV and the morphology of the sciatic nerve [20]. MNCV in nondiabetic rats was increased with increasing age, whereas it was significantly decreased in diabetic rats. Daily administration of NAC dose-dependently improved the decreased MNCV in diabetic rats.

Administration of NAC even after the development of nerve dysfunction inhibited the further lowering of MNCV. Furthermore, the effects of NAC on delayed MNCV disappeared when the drug was ceased to administer. These results indicate that NAC administration has not only a preventive effect but also a therapeutic effect on diabetic polyneuropathy. NAC administration also inhibited morphological changes in the peripheral nerve, that is, it prevented fiber atrophy and decreased fiber density of myelinated nerve fibers in diabetic rats [20]. Frequencies of abnormal fibers in teased fiber studies were also less in NAC-treated rats compared with untreated rats [20].

With these beneficial effects on neuropathic changes, NAC administration improved enhanced TNF-$\alpha$ production, increased serum peroxide and decreased glutathione content in the erythrocytes [20]. However, NAC did not affect metabolic changes such as increased glucose and sorbitol content and decreased cyclic adenosine monophosphate (cAMP) in nerve tissues [20]. It is therefore likely that the beneficial effects of NAC on diabetic nerve might be a result of inhibition of vascular and nerve damage caused by increased TNF-$\alpha$ and free radicals.

### Inhibitory Effects of Clinical Agents on TNF-$\alpha$ Production and Experimental Diabetic Neuropathy

### *Inhibition of TNF-$\alpha$ Production With Various Clinical Agents*

Because NAC, a TNF-$\alpha$ suppressant, inhibited diabetic polyneuropathy in type 1 diabetic animal models, we screened various clinical agents for TNF-$\alpha$ production in vitro and in vivo. We found inhibitory effects on TNF-$\alpha$ of nicotinamide [27], angiotensin-converting enzyme (ACE) inhibitors [28], certain calcium channel blockers, and an alpha 1 receptor blocker [29]. Among the hypoglycemic agents for diabetes treatment, gliclazide (but not glibenclamide), sulfonylurea derivatives, and troglitazone (an insulin-sensitizing thiazolidinedione), had inhibitory effects on TNF-$\alpha$ production [30].

We chose gliclazide and troglitazone (troglitazone was withdrawn from the market in March 2000 because of idiosyncratic hepatotoxicity) for the treatment of experimental diabetic neuropathy because these oral hypoglycemic agents were commonly used for diabetic patients. It has already been indicated that an ACE inhibitor ameliorated peripheral neuropathy in diabetic patients [31] and that clinical drugs that have TNF-$\alpha$–suppressing potentials have beneficial effects on insulin resistance [2].

### *Inhibition of Experimental Diabetic Neuropathy With Antidiabetic Agents With TNF-$\alpha$ Suppressor Activity*

*Gliclazide.* We studied the effects of antidiabetic agents that suppress the TNF-$\alpha$ production. Streptozotocin-induced diabetic rats were fed regular chow mixed with gliclazide or glibenclamide. MNCV and TNF-$\alpha$ production were periodically measured and histologically observed at end nerve tissues [21]. Compared with nondiabetic rats, blood glucose levels (of ~300 mg/dL, 16.7 mM) increased 3 times, whereas serum insulin levels decreased to 1/3 in diabetic rats. Neither gliclazide nor glibenclamide affected blood glucose or insulin levels in nondiabetic and diabetic rats. TNF-$\alpha$ production was enhanced in diabetic rats compared with that in nondiabetic rats, and the enhanced TNF-$\alpha$ production was suppressed in the gliclazide-treated, but not the glibenclamide-treated, rats. Increased serum peroxide levels in diabetic rats were also significantly inhibited with gliclazide but not with glibenclamide. Under these conditions, gliclazide significantly inhibited the lowering of MNCV and the increase in myelinated fiber density in diabetic rats, whereas glibenclamide did not ameliorate these abnormalities [21]. Thus, it is conceivable that gliclazide is beneficial for neuropathic changes via inhibition of TNF-$\alpha$ production and lipid oxidation in a diabetic state.

*Troglitazone.* As was the case of NAC [20] and gliclazide [21], we showed that troglitazone improved lowered MNCV and the abnormal morphology of peripheral nerves in diabetic rats irrespective of blood glucose levels [22]. The effects of troglitazone were associated with inhibition of enhanced TNF-$\alpha$ production and increased lipid oxidation in serum and nerve tissues [22].

*Pentoxifylline.* Pentoxifylline, which was known to inhibit TNF-$\alpha$ production and its action [32], also inhibited increased TNF-$\alpha$ production and lowering of MNCV in diabetic rats [23].

### MECHANISMS OF ACTION OF TNF-$\alpha$ SUPPRESSANTS IN THE INHIBITION OF DIABETIC POLYNEUROPATHY

Macrophages, which are activated by high glucose [8], oxidative stress [9], and AGEs [10] in a diabetic state, may infiltrate into nerve tissues [33] and locally produce much TNF-$\alpha$, resulting in endothelial and nerve fiber damage. In addition, it has been proposed that TNF-$\alpha$ stimulates the expression of specific proteins relevant to cellular damage, such as aldose reductase [34], protein kinase C [35], mitogen-activated protein (MAP) kinase [36] and inducible nitric oxide synthase [14, 37], all of which potentially play a role in the pathogenesis of diabetic polyneuropathy [38]. During the pathologic process, TNF-$\alpha$ may initiate and promote the development of nerve dysfunction via various pathways. Microvascular damage elicited by TNF-$\alpha$ may cause nerve ischemia and increased vascular permeability, thus permitting exposure of harmful substances to nerve fibers. On the other hand, it is shown that local TNF-$\alpha$ exerts demyelination by attacking Schwann cells [39].

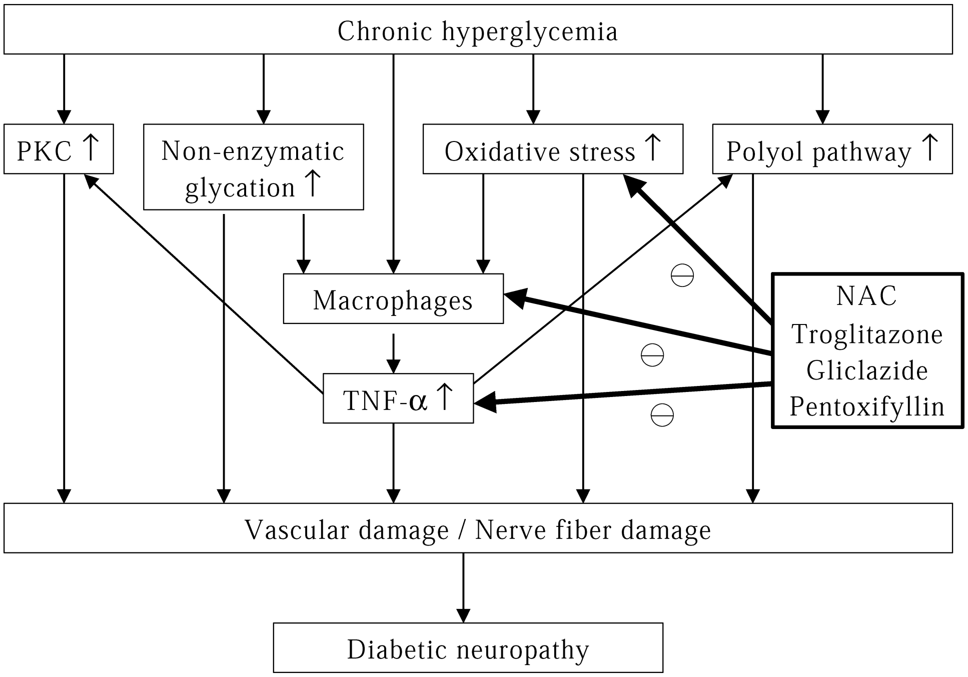

**FIGURE 2**

Hypothetical role of TNF-$\alpha$ in the pathogenesis of diabetic neuropathy and possible mechanisms of TNF-$\alpha$ suppressants in inhibition of diabetic neuropathy.

However, precise mechanisms of how TNF-$\alpha$ is responsible for the pathogenesis of diabetic polyneuropathy remain to be elucidated. It is hypothesized that NAC, gliclazide, troglitazone, and pentoxifyllin may have inhibited diabetic polyneuropathy by suppressing TNF-$\alpha$ production and also possibly by scavenging free radicals. The hypothetical mechanisms of TNF-$\alpha$ suppressants in inhibiting diabetic polyneuropathy are shown in Figure 2.

### TNF-$\alpha$ PROMOTER GENE POLYMORPHISMS AND DIABETIC POLYNEUROPATHY

The human TNF-$\alpha$ gene is located in the short arm of chromosome 6 and TNF-$\alpha$ production is genetically influenced [40]. It has been reported that there are TNF-$\alpha$ promoter gene polymorphisms that affect TNF-$\alpha$ production and that the polymorphisms are associated with insulin resistance, autoimmune diseases, and susceptibility to infectious diseases [41]. In addition, it has been suggested that there is an association of TNF-$\alpha$ restriction fragment length polymorphism (RFLP) with severity of diabetic retinopathy [42]. Recently, Higuchi and his associates found a high frequency ($\sim$15%) of the novel TNF-$\alpha$ promoter gene polymorphisms in Japanese populations [43]. Based on this report, we examined the relationship between the novel polymorphisms and diabetic neuropathy and demonstrated that the TNF-$\alpha$ promoter gene polymorphism, C($-$857)T, is significantly associated with prolonged F-wave latency in the median nerve, that is a sensitive marker of peripheral nerve dysfunction, in patients with type 2 diabetes [44]. These circumferential evidence warrants the further evaluation of the role of TNF-$\alpha$ gene in clinical manifestation of polyneuropathy in diabetic patients.

### TNF-$\alpha$ SUPPRESSANTS AND CLINICAL DIABETIC POLYNEUROPATHY

Because beneficial effects of TNF-$\alpha$ suppressants on diabetic polyneuropathy were confirmed in animal models [20–23], we were interested in exploring the clinical effects of these suppressants. We previously reported the results of a questionnaire study on diabetic polyneuropathy in approximately 33,000 diabetic patients in the Tohoku area of Japan [45]. Among these, 1228 patients were treated with troglitazone. To observe the effect of troglitazone on diabetic polyneuropathy, we retrospectively analyzed the data from the questionnaire. As shown in Figure 3, the patients treated with troglitazone had fewer subjective symptoms such as paresthesia, leg cramp, and diarrhea or constipation than those not treated with troglitazone under the similar glycemic control. This result may imply that troglitazone may be effective in ameliorating the symptoms of diabetic polyneuropathy.

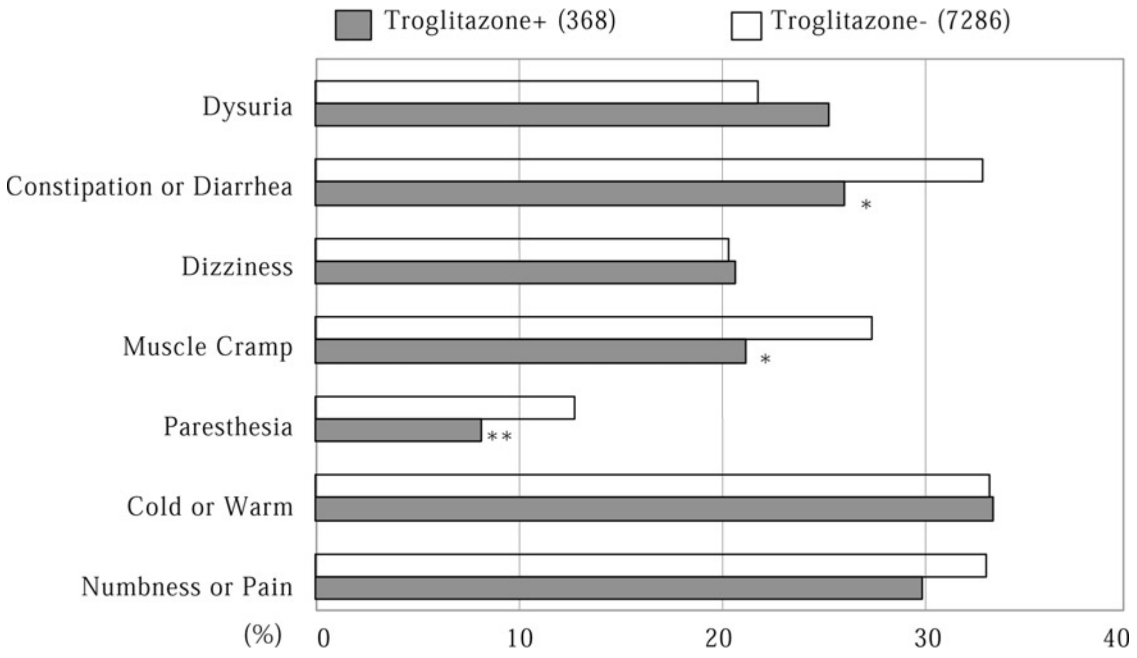

**FIGURE 3**
Frequency of symptoms of neuropathy in diabetic patients. The data of the questionnaire survey performed in Japan in 1998 [45] were subanalyzed. The patients treated with troglitazone had less frequency of symptoms of diabetic neuropathy. $^{*}p < .05$; $^{**}p < .01$

### FUTURE DIRECTION

From recent experimental and clinical studies, we consider that augmented expression and production of TNF-α are strongly implicated in the pathogenesis of diabetic polyneuropathy and suppressants of TNF-α are beneficial for alleviating signs and symptoms of this intractable disorder. However, the precise mechanism of TNF-α in the pathogenesis of diabetic polyneuropathy is largely unknown. For the future clinical application of TNF-α suppressants in effective ways, further studies are necessary to verify the role of TNF-α, for example, by evaluating (1) the effects of TNF-α administration on nerve blood flow as well as MNCV, and (2) the effects of specific inhibition of TNF-α on nerve blood flow and MNCV by using specific antibodies or specific agents. TNF-α or TNF-α receptor knockout animals may be also of use for this purpose. If the active role of TNF-α is confirmed, anti–TNF-α therapy will be valuable as a fundamental for the treatment of diabetic complications such as polyneuropathy.

### REFERENCES

[1] Rabinovitch, A. (1994) Immunoregulatory and cytokine imbalances in the pathogenesis of IDDM. Therapeutic intervention by immunostimulation? *Diabetes,* **43,** 613–621.
[2] Hotamisligil, G. S., and Spiegelman, B. M. (1994) Tumor necrosis factor alpha: A key component of the obesity-diabetes link. *Diabetes,* **43,** 1271–1278.
[3] Vlassara, H. (1990) Chronic diabetic complications and tissue glycosylation. Relevant concern for diabetes-prone black population. *Diabetes Care,* **13,** 1180–1185.
[4] King, G. L., and Brownlee, M. (1996) The cellular and molecular mechanisms of diabetic complications. *Endocrinol. Metab. Clin. North. Am.,* **25,** 255–270.
[5] Satoh, J., Seino, H., Abo, T., Tanaka, S., Shintani, S., Ohta, S., Tamura, K., Sawai, T., Nobunaga, T., Oteki, T., Kumagai, K., and Toyata, T. (1989) Recombinant human tumor necrosis factor alpha suppresses autoimmune diabetes in nonobese diabetic mice. *J. Clin. Invest.,* **84,** 1345–1348.
[6] Tanaka, S., Seino, H., Satoh, J., Fujii, N., Rikiishi, H., Zhu, X. P., Takahashi, K., Sagara, M., Nobunaga, T., and Toyota, T. (1992) Increased in vivo production of tumor necrosis factor after development of diabetes in nontreated, long-term diabetic BB rats. *Clin. Immunol. Immunopathol.,* **62,** 258–263.
[7] Katsuki, A., Sumida, Y., Murashima, S., Murata, K., Takarada, Y., Ito, K., Fujii, M., Tsuchihashi, K., Goto, H., Nakatani, K., and Yano, Y. (1998) Serum levels of tumor necrosis factor-alpha are increased in obese patients with noninsulin-dependent diabetes mellitus. *J. Clin. Endocrinol. Metab.,* **83,** 859–862.
[8] Morohoshi, M., Fujisawa, K., Uchimura, I., and Numano, F. (1996) Glucose-dependent interleukin 6 and tumor necrosis factor production by human peripheral blood monocytes in vitro. *Diabetes,* **45,** 954–959.
[9] Mendez, C., Garcia, I., and Maier, R. V. (1996) Oxidants augment endotoxin-induced activation of alveolar macrophages. *Shock,* **6,** 157–163.
[10] Vlassara, H., Brownlee, M., Manogue, K. R., Dinarello, C. A., and Pasagian, A. (1988) Cachectin/TNF and IL-1 induced by glucose-modified proteins: Role in normal tissue remodeling. *Science,* **240,** 1546–1548.

[11] Fernandez-Real, J. M., and Ricart, W. (1999) Insulin resistance and inflammation in an evolutionary perspective: The contribution of cytokine genotype/phenotype to thriftiness. *Diabetologia,* **42,** 1367–1374.

[12] Peraldi, P., Hotamisligil, G. S., Buurman, W. A., White, M. F., and Spiegelman, B. M. (1996) Tumor necrosis factor (TNF)-alpha inhibits insulin signaling through stimulation of the p55 TNF receptor and activation of sphingomyelinase. *J. Biol. Chem.,* **271,** 13018–13022.

[13] Hotamisligil, G. S., Arner, P., Caro, J. F., Atkinson, R. L., and Spiegelman, B. M. (1995) Increased adipose tissue expression of tumor necrosis factor-alpha in human obesity and insulin resistance. *J. Clin. Invest.,* **95,** 2409–2415.

[14] Ferro, T. J., Gertzberg, N., Selden, L., Neumann, P., and Johnson, A. (1997) Endothelial barrier dysfunction and p42 oxidation induced by TNF-alpha are mediated by nitric oxide. *Am. J. Physiol.,* **272(5 Pt 1),** L979–L988.

[15] Samad, F., Uysal, K. T., Wiesbrock, S. M., Pandey, M., Hotamisligil, G. S., and Loskutoff, D. J. (1999) Tumor necrosis factor alpha is a key component in the obesity-linked elevation of plasminogen activator inhibitor 1. *Proc. Natl. Acad. Sci. U.S.A.,* **96,** 6902–6907.

[16] Barone, F. C., Arvin, B., White, R. F., Miller, A., Webb, C. L., Willette, R. N., Lysko, P. G., and Feuerstein, G. Z. (1997) Tumor necrosis factor-alpha. A mediator of focal ischemic brain injury. *Stroke,* **28,** 1233–1244.

[17] Meager, A. (1999) Cytokine regulation of cellular adhesion molecule expression in inflammation. *Cytokine Growth Factor Rev.,* **10,** 27–39.

[18] Otsuki, M., Hashimoto, K., Morimoto, Y., Kishimoto, T., and Kasayama, S. (1997) Circulating vascular cell adhesion molecule-1 (VCAM-1) in atherosclerotic NIDDM patients. *Diabetes,* **46,** 2096–2101.

[19] Satoh, J., Qiang, X., Sagara, M., and Toyota, T. (1998) Treatment of diabetic neuropathy with antioxidants/TNF-$\alpha$ suppressants. *Gendaiiryo,* **30,** 55–62 (Japanese).

[20] Sagara, M., Satoh, J., Wada, R., Yagihashi, S., Takahashi, K., Fukuzawa, M., Muto, G., Muto, Y., and Toyota, T. (1996) Inhibition with *N*-acetylcysteine of development of peripheral neuropathy in streptozotocin-induced diabetic rats. *Diabetologia,* **39,** 263–269.

[21] Qiang, X., Satoh, J., Sagara, M., Fukuzawa, M., Masuda, T., Miyaguchi, S., Takahashi, K., and Toyota, T. (1998) Gliclazide inhibits diabetic neuropathy irrespective of blood glucose levels in streptozotocin-induced diabetic rats. *Metabolism,* **47,** 977–981.

[22] Qiang, X., Satoh, J., Sagara, M., Fukuzawa, M., Masuda, T., Sakata, Y., Muto, G., Muto, Y., Takahashi, K., and Toyota, T. (1998) Inhibitory effect of troglitazone on diabetic neuropathy in streptozotocin-induced diabetic rats. *Diabetologia,* **41,** 1321–1326.

[23] Qiang, X., Satoh, J., Sagara, M., Muto, G., Muto, Y., Miyaguchi, S., and Toyota, T. (1998) Treatment of diabetic neruropathy with pentoxifylline. *Tounyoubyou,* **Supple 1,** 378 (Japanese).

[24] Sen, C. K. (1998) Redox signaling and the emerging therapeutic potential of thiol antioxidants. *Biochem. Pharmacol.,* **55,** 1747–1758.

[25] Peristeris, P., Clark, B. D., Gatti, S., Faggioni, R., Mantovani, A., Mengozzi, M., Orencole, S. F., Sironi, M., and Ghezzi, P. (1992) *N*-acetylcysteine and glutathione as inhibitors of tumor necrosis factor production. *Cell. Immunol.,* **140,** 390–399.

[26] Sagara, M., Satoh, J., Zhu, X.-P., Takahashi, K., Fukuzawa, M., Muto, G., Muto, Y., and Toyota T. (1994) Inhibition with *N*-acetylcysteine of enhanced production of tumor necrosis factor in streptozotocin-induced diabetic rats. *Clin. Immunol. Immunopathol.,* **71,** 333–337.

[27] Fukuzawa, M., Satoh, J., Muto, G., Muto, Y., Nishimura, S., Miyaguchi, S., Qiang, X. L., and Toyota, T. (1997) Inhibitory effect of nicotinamide on in vitro and in vivo production of tumor necrosis factor-$\alpha$. *Immunol. Lett.,* **59,** 7–11.

[28] Fukuzawa, M., Satoh, J., Sagara, M., Muto, G., Muto, Y., Nishimura, S., Miyaguchi, S., Qiang, X. L., Sakata, Y., Nakazawa, T., Ikehata, F., Ohta, S., and Toyota, T. (1997) Angiotensin converting enzyme inhibitors suppress production of tumor necrosis factor-$\alpha$ in-vitro and in-vivo. *Immunopharmcology,* **36,** 49–55.

[29] Fukuzawa, M., Satoh, J., Ohta, S., Takahashi, K., Miyaguchi, S., Qiang, X., Sakata, Y., Nakazawa, T., Takizawa, Y., and Toyota, T. (2000) Modulation of tumor necrosis factor-$\alpha$ production with anti-hypertensive drugs. *Immunopharmacology,* **48,** 65–74.

[30] Fukuzawa, M., Satoh, J., Qiang, X., Miyaguchi, S., Sakata, Y., Nakazawa, T., Ikehata, F., Ohta, S., and Toyata, T. (1999) Inhibition of tumor necrosis factor-$\alpha$ with anti-diabetic agents. *Diabetes Res. Clin. Pract.,* **43,** 147–154.

[31] Malik, R. A., Williamson, S., Abbott, C., Carrington, A. L., Iqbal, J., Schady, W., and Boulton, A. J. (1998) Effect of angiotensin-converting-enzyme (ACE) inhibitor trandolapril on human diabetic neuropathy: Randomised double-blind controlled trial. *Lancet,* **352,** 1978–1981.

[32] Mandell, G. L. (1995) Cytokines, phagocytes, and pentoxifylline *J. Cardiovasc. Pharmacol.,* **25 (Suppl 2),** S20–S22.

[33] Carrington, A. L., and Litchfield, J. E. (1999) The aldose reductase pathway and nonenzymatic glycation in the pathogenesis of diabetic neuropathy: A critical review for the end of the 20th century. *Diabetes Rev.,* **7,** 275–299.

[34] Iwata, T., Sato, S., Jimenez, J., McGowan, M., Moroni, M., Dey, A., Ibaraki, N., Reddy, V. N., and Carper, D. (1999) Osmotic response element is required for the induction of aldose reductase by tumor necrosis factor-alpha. *J. Biol. Chem.,* **274,** 7993–8001.

[35] Magnuson, D. K., Maier, R. V., and Pohlman, T. H. (1989) Protein kinase C: A potential pathway of endothelial cell activation by endotoxin, tumor necrosis factor, and interleukin-1. *Surgery,* **106,** 216–223.

[36] Igarashi, M., Wakasaki, H., Takahara, N., Ishii, H., Jiang, Z. Y., Yamauchi, T., Kuboki, K., Meier, M., Rhodes, C. J., and King, G. L. (1999) Glucose or diabetes activates p38 mitogen-activated protein kinase via different pathways. *J. Clin. Invest.,* **103,** 185–195.

[37] Munoz-Fernandez, M. A., and Fresno, M. (1998) The role of tumour necrosis factor, interleukin 6, interferon-gamma and inducible nitric oxide synthase in the development and pathology of the nervous system. *Prog. Neurobiol.,* **56,** 307–340.

[38] Sima, A. A., and Sugimoto, K. (1999) Experimental diabetic neuropathy: An update. *Diabetologia,* **42,** 773–788.

[39] Creange, A., Barlovatz-Meimon, G., and Gherardi, R. K. (1997) Cytokines and peripheral nerve disorders. *Eur. Cytokine Netw.,* **8,** 145–151.

[40] Wilson, A. G., Symons, J. A., McDowell, T. L., McDevitt, H. O., and Duff, G. W. (1997) Effects of a polymorphism in the human tumor necrosis factor alpha promoter on transcriptional activation. *Proc. Natl. Acad. Sci. U.S.A.,* **94,** 3195–3199.

[41] Allen, R. D. (1999) Polymorphism of the human TNF alpha promoter—random variation or functional diversity? *Mol. Immunol.,* **36,** 1017–1027.

[42] Hawrami, K., Hitman, G. A., Rema, M., Snehalatha, C., Viswanathan, M., Ramachandran, A., and Mohan, V. (1996) An association in non-insulin-dependent diabetes mellitus subjects between susceptibility to retinopathy and tumor necrosis factor polymorphism. *Human Immunol.,* **46,** 49–54.

[43] Higuchi, T., Seki, N., Kamizono, S., Yamada, A., Kimura, A., Kato, H., and Itoh, K. (1998) Polymorphism of the 5′-flanking region of the human tumor necrosis factor (TNF)-alpha gene in Japanese. *Tissue Antigens,* **51,** 605–612.

[44] Housai, T., Bando, S., Takahashi, K., Negoro, K., Takizawa, Y., Ohta, S., Sekikawa, A., Hirai, M., Hinokio, Y., Suzuki, S., Satoh, J., and Toyota, T. (2001) Association of TNF-$\alpha$ promoter polymorphism C(-857)T with F-wave latency as an early marker of diabetic polyneuropathy in type 2 diabetic patients. *Diabetes,* **(Suppl 2),** A186.

[45] Satoh, J., Watanabe, T., Yagihashi, S., Tsuji, I., and Toyota, T. (2001) Diabetic survey: The questionnaire of 30,000 diabetic patients in the Tohoku area in Japan, In: *Diabetic Neuropathy; Polyol Pathway and Recent Advance*, Edited by Matsuoka, K., Hotta, N., and Yagihashi, S., pp. 293–300. Tokyo, Gendaiiryosya (Japanese).

